# EV68-228-N monoclonal antibody treatment halts progression of paralysis in a mouse model of EV-D68 induced acute flaccid myelitis

**DOI:** 10.1128/mbio.03906-24

**Published:** 2025-03-24

**Authors:** Michael J. Rudy, Courtney J. Wilson, Brendan Hinckley, Danielle C. Baker, Joshua M. Royal, Marshall P. Hoke, Miles B. Brennan, Matthew R. Vogt, Penny Clarke, Kenneth L. Tyler

**Affiliations:** 1 Department of Neurology, University of Colorado, Aurora, Colorado, USA; 2 Department of Research and Discovery, KBio Inc., Owensboro, Kentucky, USA; 3 Department of Regulatory Affairs, ZabBio Inc., San Diego, California, USA; 4 Borrasca Consulting LLC, Denver, Colorado, USA; 5 Departments of Pediatrics and Microbiology and Immunology, University of North Carolina at Chapel Hill School of Medicine, Chapel Hill, North Carolina, USA; 6 Department of Immunology and Microbiology, University of Colorado, Aurora, Colorado, USA; 7 Division of Infectious Disease, Department of Medicine, University of Colorado, Aurora, Colorado, USA; Duke University School of Medicine, Durham, North Carolina, USA

**Keywords:** Enterovirus D-68, acute flaccid myelitis, treatment, monoclonal antibodies

## Abstract

In 2014, 2016, and 2018, infection with enterovirus D68 (EV-D68) was associated with outbreaks of a poliomyelitis-like paralytic syndrome, called acute flaccid myelitis (AFM). While only a small fraction of patients infected with EV-D68 developed AFM, this subgroup of patients does not typically seek treatment until after the onset of neurological symptoms. There are currently no approved human monoclonal antibody therapies or vaccines available for EV-D68. Here, we show that a monoclonal antibody, EV68-228-N, can quickly stop the progression of paralysis in a mouse model of AFM, even when treatment is initiated after the onset of paralysis. We found that EV68-228-N effectively halted the progression of paralysis when tested against both 2014 and 2016 EV-D68 isolates in an immunocompetent mouse model of AFM. All animal experiments were conducted in a blinded fashion. The IC_50_ of EV68-228-N against 2014 and 2016 EV-D68 isolates was confirmed *in vitro* to be less than 330 ng/mL, and EV68-228-N was found to be equally effective at neutralizing 2018 and 2022 viral isolates without any evidence of emerging resistance. We further show that, following infection with EV-D68, mice treated with EV68-228-N have more surviving motor neurons in the spinal cord’s lumbar enlargement than control treated animals. Taken together, this work suggests that EV68-228-N treatment has the potential to halt the progression of paralysis in AFM patients who present at the clinic with neurologic symptoms and that EV68-228-N will retain neutralization potential against emerging EV-D68 isolates.

**IMPORTANCE:**

Enterovirus D-68 (EV-D68) associated acute flaccid myelitis (AFM) is an emergent poliomyelitis-like illness occurring predominantly in children. There are currently no proven effective therapies. We describe the use of a human monoclonal antibody (EV68-228-N) in a murine model of EV-D68 AFM in which therapy prevents progression of paralysis even when treatment is instituted after onset of weakness.

**Clinical Trials:**

This study is registered with ClinicalTrials.gov as NCT06444048.

## INTRODUCTION

In 2014, 2016, and 2018, there were outbreaks of acute flaccid myelitis (AFM) in the United States. Many of these cases were associated with infection by enterovirus D68 (EV-D68). EV-D68 is endemic in the United States, and the prodromal illness typically includes cough, congestion, and sore throat, with some patients reporting nausea and vomiting (1). While respiratory illness can be severe and can result in hospitalization, most patients develop only mild respiratory symptoms. The most serious outcome of EV-D68 infection is rapid onset muscle weakness and paralysis in one or more limbs, known as AFM. Since 2022, EV-D68 has continued to circulate and cause respiratory disease but for unexplained reasons has not been associated with an increase in AFM cases (2). Only a small fraction of EV-D68 cases progressed to AFM, but in these patients, muscle weakness typically began 5–7 days following respiratory symptoms, with many patients reporting improvement in their preceding respiratory and systemic symptoms before the onset of neurological deficits (3). The pattern of muscle weakness and associated clinical and laboratory studies in AFM are consistent with virus-induced death of spinal cord motor neurons. Viral infection of spinal cord motor neurons has been confirmed in mouse models of EV-D68 infection (4, 5), as well as in a child with EV-D68 in his CSF who died of an AFM-like illness (6). Most patients who developed AFM have residual long-term motor deficits, with the most profoundly affected muscle groups showing little to no improvement in strength (3).

There are currently no approved antiviral therapies for treatment of AFM, nor is there an available EV-D68 vaccine (7). Studies in experimental murine models of EV-D68-induced AFM have shown that pooled human intravenous immunoglobulin (hIVIG) and anti-EV-D68 neutralizing monoclonal and polyclonal antibodies can significantly reduce paralysis and improve survival when administered prior to the onset of paralysis (8–11). Anti-EV-D68 antibodies have also been shown to slow the progression of paralysis when administered after the onset of paralysis (8). However, another monoclonal antibody tested in this model (15c5-Chmra), which was originally isolated from mice immunized with a 2014 EV-D68 viral isolate, was minimally effective against an EV-D68 isolate from the 2016 epidemic and completely ineffective against a viral isolate from the 2018 EV-D68 epidemic (8).

A promising new anti-EV-D68 monoclonal antibody (EV68-228) was identified by screening B-cells isolated from the blood of patients who had recovered from EV-D68 respiratory infection for binding potential against five modern EV-D68 isolates. Vogt et al. (9) show that a version of EV68-228 produced in mammalian cell culture has the ability to neutralize 2014 EV-D68 clades B1 and D isolates at a 50% inhibitory concentration of 5 ng/mL or less, as well as the distantly related ancestral Fermon strain at 52 ng/mL (9), suggesting that EV68-228 will have robust neutralization potential against a wide range of EV-D68 viral isolates. They further examined the efficacy of EV68-228 in an AG129 (deficient in receptors for interferon α/β and γ) adult mouse model and found that EV68-228-treated mice had improved survival and reduced neurologic disease when treatment was initiated as late as 2 days postinfection (DPI-2). However, EV-D68 infection in AG129 mice results in widespread infection of all permissive host tissues including the lung, liver, kidney, spleen, brain, blood, and spinal cord (12), as well as widespread infection of all skeletal muscles (13), and often results in death of the host between DPI-4 and DPI-6 (9).

We used an immunocompetent neonatal Swiss–Webster mouse model, in which 75% of untreated animals will survive and clear viral infection (but nearly 100% have lasting paralysis), to show that antibody treatment can effectively halt the progression of paralysis and prevent the death of motor neurons when treatment is initiated after the onset of paralysis. In this model, mice are infected with non-mouse adapted EV-D68 isolates from either the 2014 (US/IL/14-18952) or 2016 (2016-334-74) epidemics, and treatment is withheld until after the onset of moderate paralysis in a single limb. We show that human monoclonal antibody EV68-228 produced in *Nicotiana benthamiana* (EV68-228-N), which has an Fab fragment matching that published for the monoclonal antibody EV68-228 (9), can effectively halt the progression of paralysis when administered after the onset of paralysis (DPI-3 through DPI-6). While EV68-228-N treatment does not reverse paralysis which was present prior to treatment, it stops the progression of paralysis to the contralateral hindlimb and to the forelimbs, and it does so more quickly than pooled human IVIG. In addition, there are more surviving motor neurons in the lumbar enlargement of infected animals following EV68-228-N treatment, compared to hIVIG- and placebo-treated animals. Notably, we also demonstrate, *in vitro*, that 228 N potently neutralizes a wider array of EV-D68 isolates than previously tested (2014 EV-D68 clades D, B1, and B2), as well as isolates from 2016, 2018, and 2022.

The EV68-228-N antibody described in this paper is now entering a phase 1, randomized, double-blind, placebo-controlled study to evaluate the safety and tolerability of an EV-D68-specific monoclonal antibody in healthy adults (ClinicalTrials.gov ID NCT06444048).

## RESULTS

### Efficacy of EV68-228-N monoclonal antibody in a clinical treatment model of AFM

**Fig 1:**
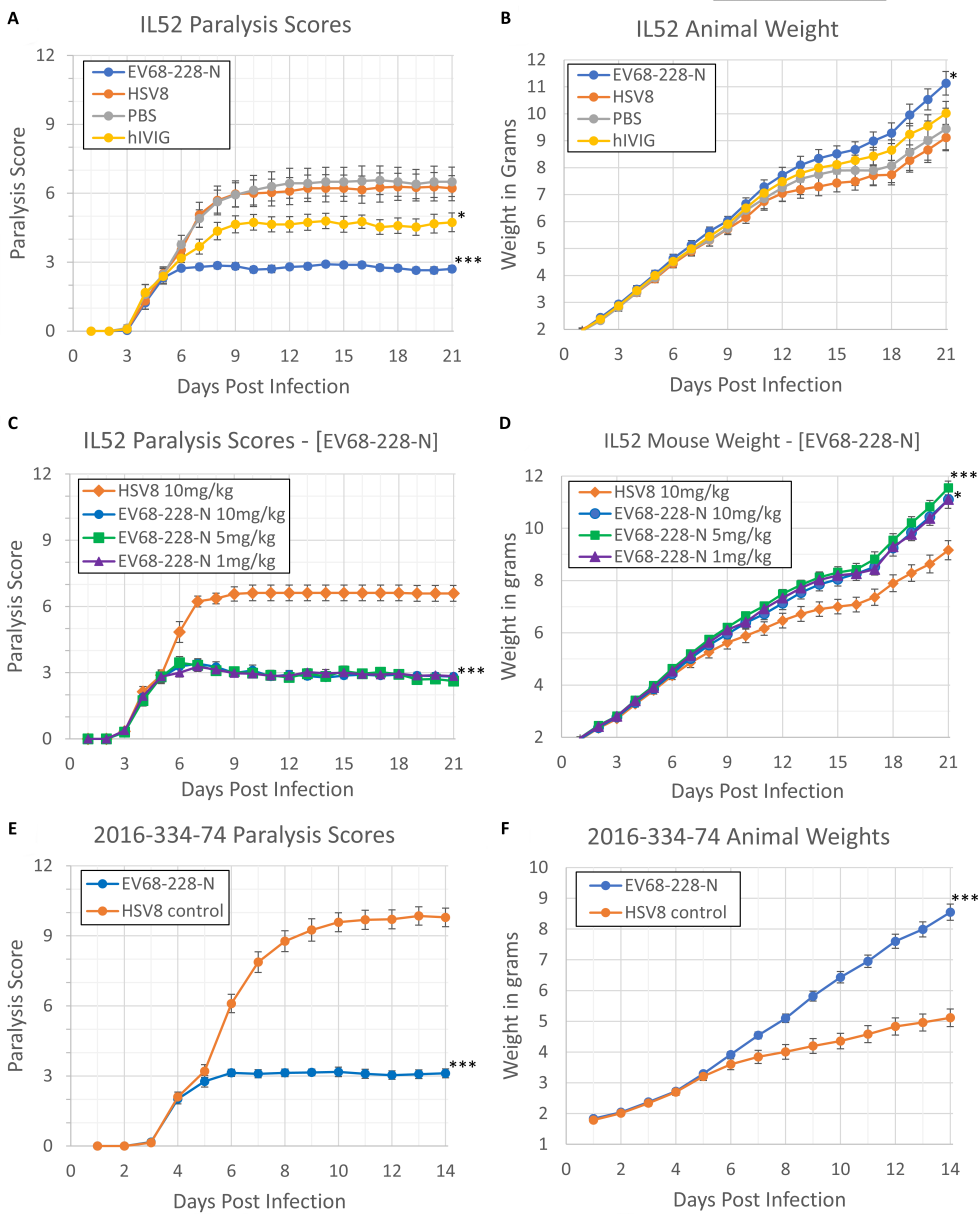
Monoclonal antibody treatment of mice infected with EV-D68 beginning when an animal had a paralysis score equal to or >1.5 (the point where paralysis was unmistakable in a mouse pup). Infected mice were treated with either EV68-228-N mAb, HSV8 mAb (non-reactive to EV-D68), hIVIG (GAMMAGARD LIQUID), or PBS. (A) Total paralysis score (0 = unparalyzed; 12 = quadriplegic) over 21-day time-course of experiment for mice infected with US/IL/14-18952. Paralysis scores were significantly different for EV68-228-N vs HSV8 (***P < 0.0001 repeated measures ANOVA), for hIVIG vs HSV8 (*P* = 0.0347 repeated measures ANOVA), and for 228 vs hIVIG (***P < 0.0001 repeated measures ANOVA). Error bars represent SEM. (B) Animal weight for same mice shown in panel A. Weight of EV68-228-N-treated group was significantly higher than HSV8-treated group (*P = 0.0447 repeated measures ANOVA). Error bars represent SEM. (C) Total paralysis score (0 = unparalyzed; 12 = quadriplegic) over 21-day time-course of experiment comparing 1-, 5-, and 10-mg/kg EV68-228-N-treated animals with 10-mg/kg HSV8-treated animals for mice infected with US/IL/14-18952. Paralysis scores were significantly different for all EV68-228-N-treated animals compared to HSV8 (*P < 0.0001 repeated measures ANOVA). Error bars represent SEM. (D) Animal weight for the same mice shown in panel C. Weight of EV68-228-N-treated group was significantly higher than HSV8-treated group at all concentrations: 228@10 mg/kg vs HSV8, *P = 0.0125 repeated measures ANOVA (days 14–21), 228@5 mg/kg vs HSV8, *P* = 0.0003 repeated measures ANOVA (days 14–21), 228@1 mg/kg vs HSV8, *P* = 0.0122 repeated measures ANOVA (days 14–21). Error bars represent SEM. (E) Total paralysis score (0 = unparalyzed; 12 = quadriplegic) over 14-day time-course of experiment for mice infected with 2016–334-74. Paralysis scores were significantly different between groups (***P < 0.0001 repeated measures ANOVA). Error bars represent SEM. (F) Animal weight for the same mice shown in panel E. Weight of EV68-228-N-treated group was significantly higher than HSV8-treated group (***P < 0.0001 repeated measures ANOVA). Error bars represent SEM.

A neutralizing human monoclonal antibody (EV68-228-N) was tested for efficacy against EV-D68-induced AFM. A human chimeric anti-herpes simplex virus human chimeric antibody-8 (HSV8) monoclonal antibody was used as a non-EV-D68 specific negative control, and pooled human IVIG (GAMMAGARD LIQUID, Takeda Pharmaceuticals) was tested as a positive control. Swiss–Webster mouse pups weighing 1.5–2.0 g (postnatal days 1 and 2) were infected with 10,000 TCID_50_ of EV-D68 (strain US/IL/14-18952 or 2016-334-74) by intramuscular injection, and individual limbs were assessed for the development of paralysis using a previously validated scoring system (10). Briefly, each limb received a paralysis score between 0 (no paralysis) and 3 (complete paralysis), with a score of 12 representing complete quadriplegia. All mice were treated in a “clinical treatment model,” where antibody is administered after the onset of paralysis to better represent the likely timing of therapeutic intervention with EV68-228-N in the clinic. We found that, while none of the treatments were able to reverse the paralysis which was present prior to treatment, the progression of paralysis was quickly stopped in EV68-228-N-treated animals (0.76 ± 0.21 days to max paralysis score in US/IL/14–18952-infected EV68-228-N-treated group compared to 4.56 ± 0.75 days to max paralysis in HSV8-treated group; *P* < 0.0001 one-way analysis of variance [ANOVA]) resulting in significantly (*P* < 0.0001 repeated measures ANOVA) lower paralysis scores at the end of the experiment compared to HSV8- or phosphate buffered saline (PBS)-treated groups and significantly (*P* < 0.0001 repeated measures ANOVA) less paralysis than even hIVIG-treated controls (Fig. 1A). Animals in the EV68-228-N-treated group also had significantly (*P* = 0.0447 repeated measures ANOVA) higher weights than HSV8-treated controls at the end of the experiment (11.13 ± 0.44 g in EV68-228-N-treated group on day 21 vs 9.18 ± 0.50 g in HSV8 treated group on day 21), suggesting that animals in the EV68-228-N group developed less severe illness than HSV8 controls (Fig. 1B). We also examined whether there was any difference between EV68-228-N mAb at 1, 5, and 10 mg/kg in terms of efficacy. We found that all concentrations of EV68-228-N mAb had a similar effect on final paralysis scores (Fig. 1C) and that the weights of animals treated with 1-, 5-, or 10-mg/kg EV68-228-N mAb were significantly (*P* = 0.0125 repeated measures ANOVA) higher than the weights of animals treated with HSV8 control mAb (Fig. 1D) (11.10 ± 0.37, 11.55 ± 0.26, and 11.11 ± 0.26 g for 1-, 5-, and 10-mg/kg groups, respectively, compared to 9.16 ± 0.37 g for HSV8-treated controls on DPI-21). We previously published results for a chimeric human–mouse monoclonal antibody, called 15c5-Chmra, which slowed paralysis progression in mice infected with a 2014 EV-D68 isolate (US/IL/14-18952) but showed reduced efficacy against a more recent 2016 EV-D68 isolate (2016-334-74) and no efficacy against a 2018 viral isolate (2018-23089) (8). To examine whether EV68-228-N would retain efficacy against more recent EV-D68 strains, we tested EV68-228-N mAb against EV-D68 isolate 2016-334-74 in our clinical model. We found that the paralysis scores of mice in the EV68-228-N-treated group were significantly (*P* < 0.0001 repeated measures ANOVA) lower than mice in the HSV8-treated control group (Fig. 1E). The weights of EV68-228-N-treated animals were also significantly (*P* < 0.0001 repeated measures ANOVA) higher than HSV8-treated controls, suggesting reduced illness in the EV68-228-N-treated group (Fig. 1F) (8.54 ± 0.26 g for EV68-228-N-treated group at DPI-14 compared to 5.11 ± 0.28 g for HSV8-treated group at DPI-14).

When mice are intramuscularly infected with the 2014 EV-D68 isolate US/IL/14-18952, paralysis initially develops in the injected hindlimb between DPI-3 and DPI-6. If untreated, paralysis will usually be observed in the contralateral hindlimb approximately 3 days later than the injected hindlimb, and in approximately 15% of animals, paralysis/weakness will be observed in the forelimbs 2 days after the contralateral hindlimb (8). The more quickly the progression of paralysis can be halted, the lower an animal’s overall paralysis score will be. Table 1 shows the average day of treatment (DPI) for mice in each treatment group, as well as the average paralysis at the time of treatment for that group. In the “clinical treatment model,” not all mice are treated on the same day of postinfection (DPI-3 through DPI-6) or at the same paralysis score (1.5 through 3). Figure S1 shows the raw data for four representative mice (two HSV8-treated and two EV68-228-N-treated mice) detailing paralysis progression by day, time of treatment, and day of treatment. The scores from all mice are averaged together to give the group average reported in Table 1. In Table 1, the column labeled “Δ paralysis score after treatment” shows how much the average paralysis score in that group increased between day of treatment and the last day of the experiment (a lower number represents a smaller increase in paralysis). Mice in the EV68-228-N (10 mg/kg)-treated group (infected with US/IL/14-18952) had an average paralysis score of 2.4 at the time of treatment and an average paralysis score of 2.8 at the end of the experiment, meaning that the paralysis score increased by only 0.4 following treatment. EV68-228-N treatment effectively prevented the spread of paralysis to the contralateral hindlimb and both forelimbs and provided superior protection from progression of paralysis compared to PBS- or HSV8-treated mice (infected with US/IL/14-18952), which saw paralysis scores increase by 4.4 and 4.1, respectively, and lost all function in the contralateral hindlimb as well as moderate weakness in one of the forelimbs following treatment. To facilitate comparison between antibody treatments, data from our previously published work (8) showing the progression of paralysis in US/IL/14-18952-infected mice treated with 15c5-Chmra in the same clinical treatment model were copied into Table 1 (data indicated by footnote *a*). The paralysis scores of hIVIG- and 15c5-Chmra-treated animals continued to increase for 4.18 ± 0.91 and 3.00 ± 0.73 days, respectively (following treatment). During this time, the paralysis scores in those groups increased by +2.2 and +2.5 points, respectively. Compare this to the paralysis scores in the EV68-228-N-treated group, which continued to increase for only 0.76 ± 0.21 days following treatment and increased by only 0.4 points during that time. Taken together, these data indicate that EV68-228-N treatment halts the progression of paralysis more quickly than either hIVIG or 15c5-Chmra treatment, leading to better paralysis outcome in the EV68-228-N-treated group.

**TABLE 1:** Experimental details for each treatment group

Treatment	Viral strain	Day of treatment	Paralysis score at time of treatment	Δ paralysis score after treatment	Number of mice in group
PBS (−) control	US/IL/14-18952	4.3 ± 0.17	2.3 ± 0.25	+4.4	*N* = 14
Anti-HSV8 (−) control	US/IL/14-18952	4.3 ± 0.14	2.3 ± 0.10	+4.1	*N* = 38
hIVIG (+) control	US/IL/14-18952	4.5 ± 0.22	2.5 ± 0.18	+2.2	*N* = 17
EV68-228-N 10 mg/kg	US/IL/14-18952	4.5 ± 0.13	2.4 ± 0.09	+0.4	*N* = 40
EV68-228-N 5 mg/kg	US/IL/14-18952	4.2 ± 0.18	2.2 ± 0.14	+0.4	*N* = 21
EV68-228-N 1 mg/kg	US/IL/14-18952	4.1 ± 0.18	2.2 ± 0.13	+0.6	*N* = 22
15c5-Chmra 15 mg/kg	US/IL/14-18952	4.0 ± 0.0^*a*^	2.0 ± 0.0^*a*^	+2.5^*a*^	*N* = 08^*a*^
EV68-228-N 10 mg/kg	2016-334-74	4.2 ± 0.15	2.3 ± 0.12	+0.7	*N* = 26
Anti-HSV8 (−) control	2016-334-74	4.2 ± 0.13	2.4 ± 0.13	+7.4	*N* = 24
15c5-Chmra 15 mg/kg	2016-334-74	4.2 ± 1.0^*a*^	2.2 ± 0.7^*a*^	+3.0^*a*^	*N* = 26^*a*^

^*a*^ *, historical data from reference 8.

When mice are intramuscularly infected with the 2016 EV-D68 isolate 2016-334-74, paralysis initially develops in the injected hindlimb between DPI-3 and DPI-6. If untreated, paralysis will spread to the ipsilateral forelimb approximately 1 day later than the injected hindlimb and to the contralateral hindlimb, approximately 2 days later than the injected hindlimb. In approximately 71% of animals, paralysis will affect all limbs within 4 days following paralysis in the injected hindlimb. Table 1 shows the average increase in paralysis for animals infected with the 2016 EV-D68 epidemic isolate (2016-334-74) and treated with EV68-228-N, non-EV-D68-specific antibody (HSV8), or 15c5-Chmra (8). Mice in the EV68-228-N (10 mg/kg)-treated group (infected with 2016-334-74) had an average paralysis score of 2.3 at the time of treatment, and following treatment, this paralysis score increased by only 0.7, indicating that EV68-228-N treatment prevents the spread of paralysis to all other limbs. In comparison, mice treated with a non-EV-D68-specific antibody (HSV8) lost all function in both hindlimbs and in the ipsilateral forelimb and showed weakness in the contralateral forelimb. The paralysis scores of EV68-228-N-treated animals also increased far less than 15c5-Chmra-treated animals (with an increase of 3.0 following treatment), despite also being treated with a lower dose of mAb (10 vs 15 mg/kg). The 15c5 mouse paralysis data shown in Table 1 was taken from our previously published data (8) and was not repeated alongside the EV68-228-N-, hIVIG-, HSV8-, and PBS-treated mice. All historical data are marked with a footnote in Table 1. No figures, besides Table 1, show historical data.

### Viral titer in the spinal cord and muscle

**Fig 2:**
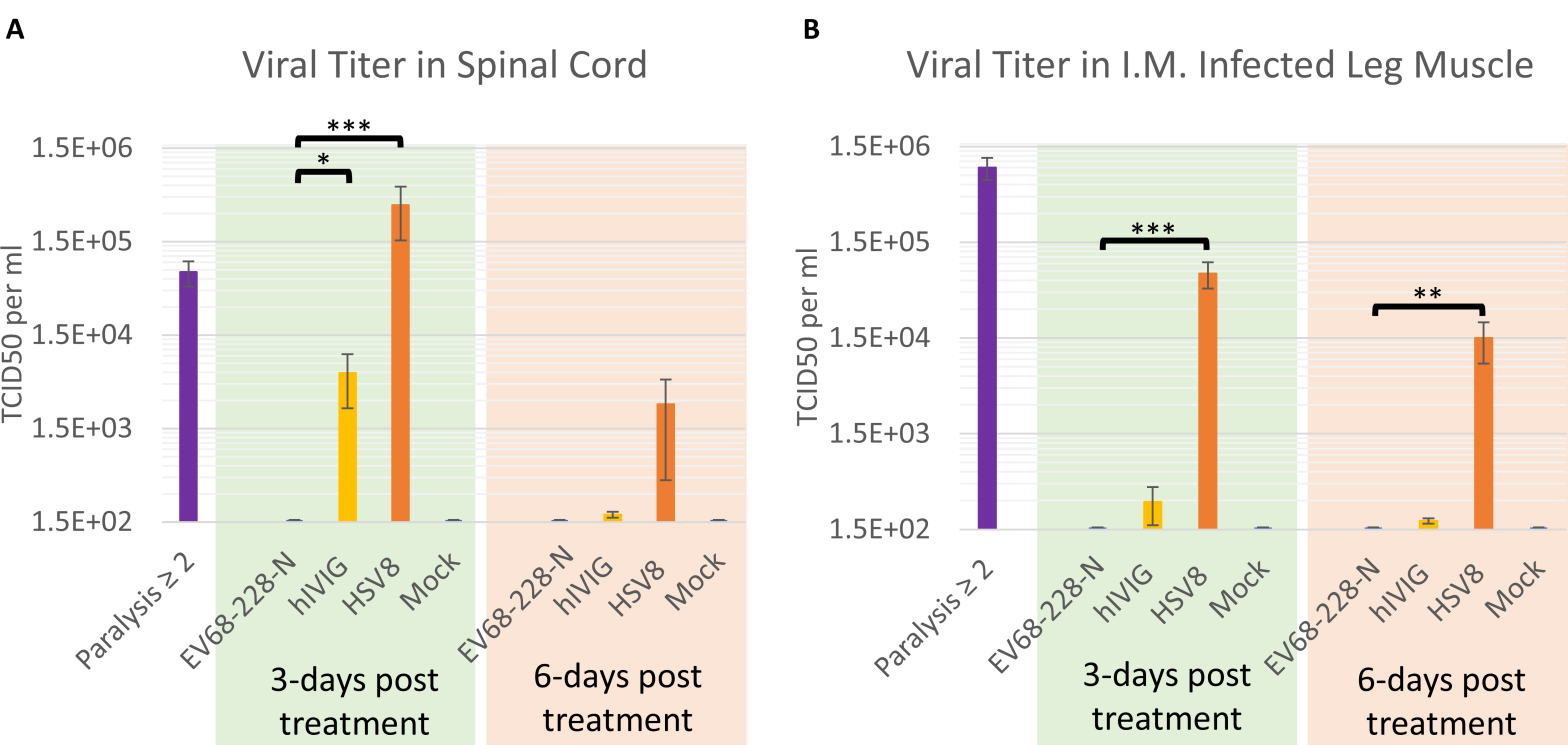
Viral titer in the spinal cord and muscle. (A) Viral titer in the spinal cord from tissue collected when paralysis score ≥ 1.5 (single purple bar represents samples from untreated animals), 3 days after treatment and 6 days after treatment. There is a significant difference in viral titer between EV68-228-N- and HSV8-treated animals at 3 days posttreatment (*P < 0.0001, one-way ANOVA and Dunnett’s method on log transformed data). There is a significant difference in spinal cord viral titer between EV68-228-N- and hIVIG-treated animals at 3 days posttreatment (*P = 0.0401, one-way ANOVA and Dunnett’s method on log transformed data). *N* = 6–8 in all groups. Error bars represent SEM. TCID_50_ values are as follows: paralysis ≥2 = 70,717 ± 21,505; 3 days posttreatment (EV68-228-N = 158 ± 0; hIVIG = 5906 ± 3444; HSV8 = 367,220 ± 213,122; mock = 158 ± 0); 6 days posttreatment (EV68-228-N = 158 ± 0; hIVIG = 179 ± 13; HSV8 = 2,719 ± 2,301; mock = 158 ± 0). Limit of detection = 158 TCID_50_ per mL. (B) Viral titer in the quadriceps muscle from tissue collected when paralysis score ≥1.5 (single purple bar represents samples from untreated animals), 3 days after treatment and 6 days after treatment. There is a significant difference in muscle viral titer between EV68-228-N- and HSV8-treated animals at 3 days posttreatment (*P < 0.0001, one-way ANOVA and Dunnett’s method on log transformed data) and at 6 days posttreatment (*P = 0.0050, one-way ANOVA and Dunnett’s method on log transformed data). *N* = 6–8 in all groups. Error bars represent SEM. TCID_50_ values are as follows: paralysis ≥2 = 904,500 ± 236,559; 3 days posttreatment (EV68-228-N = 158 ± 0; hIVIG = 292 ± 125; HSV8 = 70,717 ± 21,506; mock = 158 ± 0); 6 days posttreatment (EV68-228-N = 158 ± 0; hIVIG = 185 ± 12; HSV8 = 15,002 ± 6,882; mock = 158 ± 0). Limit of detection = 158 TCID_50_ per mL.

Viral titer was measured in the spinal cords, muscle tissue, and blood from animals infected with US/IL/14–18952 and subsequently treated with EV68-228-N, hIVIG, or HSV8, as well as uninfected controls. We found that the spinal cords of EV68-228-N-treated animals had viral titers below the limit of detection at both 3 days and 6 days posttreatment (Fig. 2A) (limit of detection = 158 TCID_50_ per mL). At 3 days posttreatment, EV68-228-N-treated animals had significantly (*P* < 0.0001, one-way ANOVA) less infectious virus in the spinal cords compared to non-specific antibody (HSV8)-treated control mice (EV68-228-N = 158 ± 0 TCID_50_ per ml; HSV8 = 367,220 ± 213,122 TCID_50_ per mL). EV68-228-N-treated animals also had significantly (*P* = 0.0401, one-way ANOVA) lower viral titer than hIVIG-treated animals at 3 days posttreatment (EV68-228-N = 158 ± 0 TCID_50_ per mL; hIVIG = 5,906 ± 3,444 TCID_50_ per mL). At 6 days posttreatment, virus was still detectable in many HSV8-treated spinal cords but was no longer significantly different from the EV68-228-N-treated group (HSV8 = 2,719 ± 2,301 TCID_50_ per mL; EV68-228-N = 158 ± 0 TCID_50_ per mL). The purple bar labeled “Paralysis ≥2” in Fig. 2A and B indicates that there were high viral titers in both the spinal cord and muscle at the time of treatment (70,717 ± 21,505 TCID_50_ per mL in the spinal cord and 904,500 ± 236,559 TCID_50_ per mL in muscle). Treatment with EV68-228-N mAb significantly (*P* < 0.0001, one-way ANOVA) reduced viral titer in the spinal cord at 3 days posttreatment (EV68-228-N = 158 ± 0 TCID_50_ per mL), and viral titers from EV68-228-N-treated animals remained below the limit of detection at DPI-6 (orange bar labeled “HSV8” in Fig. 2A) (limit of detection = 158 ± 0 TCID_50_ per mL). Similarly, we also measured viral titer in the left quadriceps muscle (the site of viral infection) and found that all animals treated with EV68-228-N mAb had viral titers below the limit of detection at 3 and 6 days posttreatment (Fig. 2B). Compared to viral titers in HSV8-treated animals, EV68-228-N-treated animals had significantly lower viral titers in the quadriceps muscle at both 3 and 6 days posttreatment (*P* < 0.0001 and *P* = 0.0050, respectively, one-way ANOVA) (HSV8 = 70,717 ± 21,506 and EV68-228-N = 158 ± 0 TCID_50_ per mL at 3 days posttreatment; HSV8 = 15,002 ± 6,882 and EV68-228-N = 158 ± 0 TCID_50_ per mL at 6 days posttreatment). As expected, viral titers from mock infected animals were below the limit of detection at all time points in both the spinal cord and muscle. Viral titer was also assessed in blood and contralateral muscle collected from all animals shown in Fig. 2A and B; however, no detectible virus was ever found in the blood of any animal from any group, and viral titer was rarely detected in the contralateral quadriceps muscle (data not shown). The lack of viral titer in both the spinal cord and muscle by TCID_50_ assay (Fig. 2), as well as PCR data showing that viral RNA was undetectable in the spinal cord and muscle at both DPI-3 and DPI-6 (Fig. S2), indicates that EV68-228-N treatment is completely eliminating virus from these tissues and not driving virus into latency in the spinal cord or muscle tissue, to reemerge when antibody levels drop. We also reassessed paralysis in mice from the treatment and control groups at DPI-56 and did not see a significant change (either improvement or worsening) in paralysis scores in either group (data not shown).

### Histology in the spinal cord

**Fig 3:**
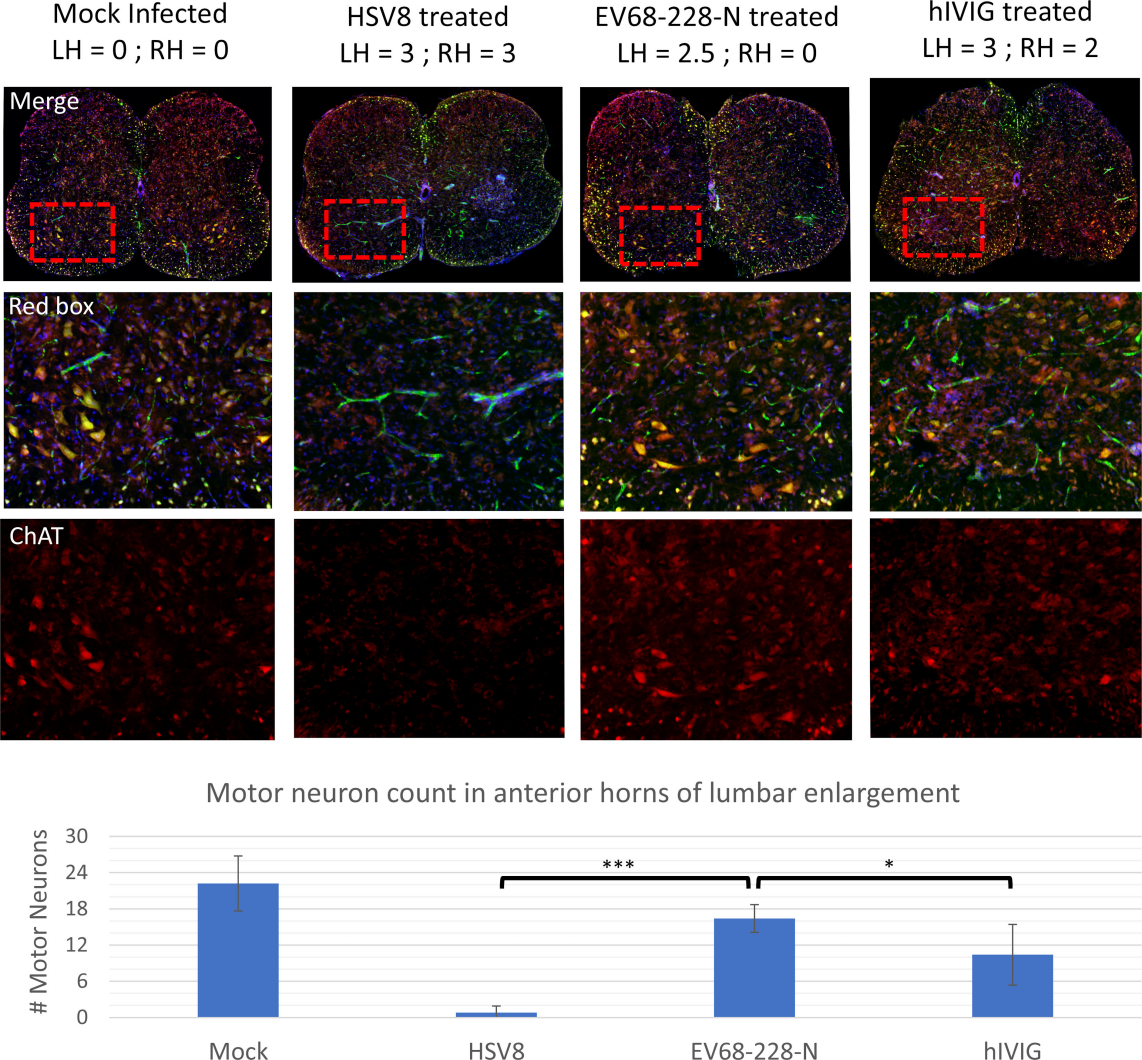
Immunofluorescence of 20-µm transverse section through lumbar enlargement of spinal cords collected from DPI-8 mice. Mock animals were not infected with virus. HSV8 (negative control), EV68-228-N, and hIVIG (positive control) were all infected with US/IL/14-18952 prior to antibody treatment. Paralysis scores of mice are recorded at the top of the column. “LH” is paralysis in the left hindlimb and “RH” is paralysis in the right hindlimb. Paralysis scores range from 0 = unparalyzed to 3 = complete paralysis for each limb. Top image is a representative section through lumbar enlargement of the spinal cord where the red channel shows ChAT staining, the green channel shows NeuN staining, and the blue channel shows DAPI staining of nucleus. Red box surrounds the ventral horn on the right side of the spinal cord and is shown as enlargement in the middle row of IHC images. Motor neuron-specific stain (ChAT) is shown in the bottom row. The bar graph shows average motor neuron counts from five serial sections through the lumbar enlargement (spaced 200-µm apart) of a single animal in each treatment group. One-way ANOVA with post hoc Dunnett’s method comparing all groups to EV68-228-N where HSV8 vs EV68-228-N is significant (***P < 0.0001) and hIVIG vs EV68-228-N is significant (*P = 0.0475). Error bars represent standard deviation. Average motor neuron numbers are as follows: mock = 22.2 ± 4.5; HSV8 = 0.8 ± 1.1; EV68-228-N = 16.4 ± 2.3; hIVIG = 10.4 ± 5.0.

Spinal cords were collected from US/IL/14–18952-infected animals treated with EV68-228-N mAb, HSV8 mAb, and hIVIG, as well as from an uninfected control mouse. Immunohistochemical (IHC) staining of coronal sections of the lumbar enlargement for choline acetyltransferase (ChAT; motor neuron specific) and neuronal nuclei (NeuN; stains all neurons) showed that motor neurons are present in the ventral horn of a mock infected animal (Fig. 3, “mock infected” column, average number of motor neurons in mock infected = 22.2 ± 4.5). The HSV8-treated animal with complete paralysis of both hindlimbs had lost almost all motor neurons from the ventral horn of the lumbar enlargement (Fig. 3, “HSV8 mAb-treated” column) (HSV8 = 0.8 ± 1.1 motor neurons), while motor neurons were still present in the same region from the EV68-228-N-treated animal that still had complete function in the right hindlimb and a small amount of movement in the left hindlimb (Fig. 3 “228 mAb-treated” column) (EV68-228-N = 16.4 ± 2.3 motor neurons). Treatment with hIVIG also increased the number of surviving motor neurons (Fig. 3, “hIVIG-treated” column), compared to control treated mice (hIVIG = 10.4 ± 5.0 motor neurons vs HSV8 = 0.8 ± 1.1 motor neurons). One-way ANOVA with post hoc Dunnett’s method comparing all groups to EV68-228-N found that there were significantly more motor neurons throughout the lumbar enlargement of an EV68-228-N-treated animal compared to animals in either HSV8-treated (***P < 0.0001) or hIVIG-treated (*P = 0.0475) groups (EV68-228-N = 16.4 ± 2.3 motor neurons; hIVIG = 10.4 ± 5.0 motor neurons; HSV8 = 0.8 ± 1.1 motor neurons). Statistics were based on five serial sections collected at 200-µm intervals throughout the lumbar enlargement of one animal in each group. Immunofluorescent analysis of the lumbar enlargement of 21 animals with paralysis scores between 0 and 6 indicates that there is a strong (*r*^2^ = 0.96) linear correlation between paralysis score in the hindlimbs and motor neuron count in mice (data not shown). Therefore, the differences in paralysis scores between the groups shown in Fig. 1 are a behavioral readout of the number of motor neurons that remain in the spinal cords of these animals.

### Neutralization potency of EV68-228-N *in vitro*

**Fig 4:**
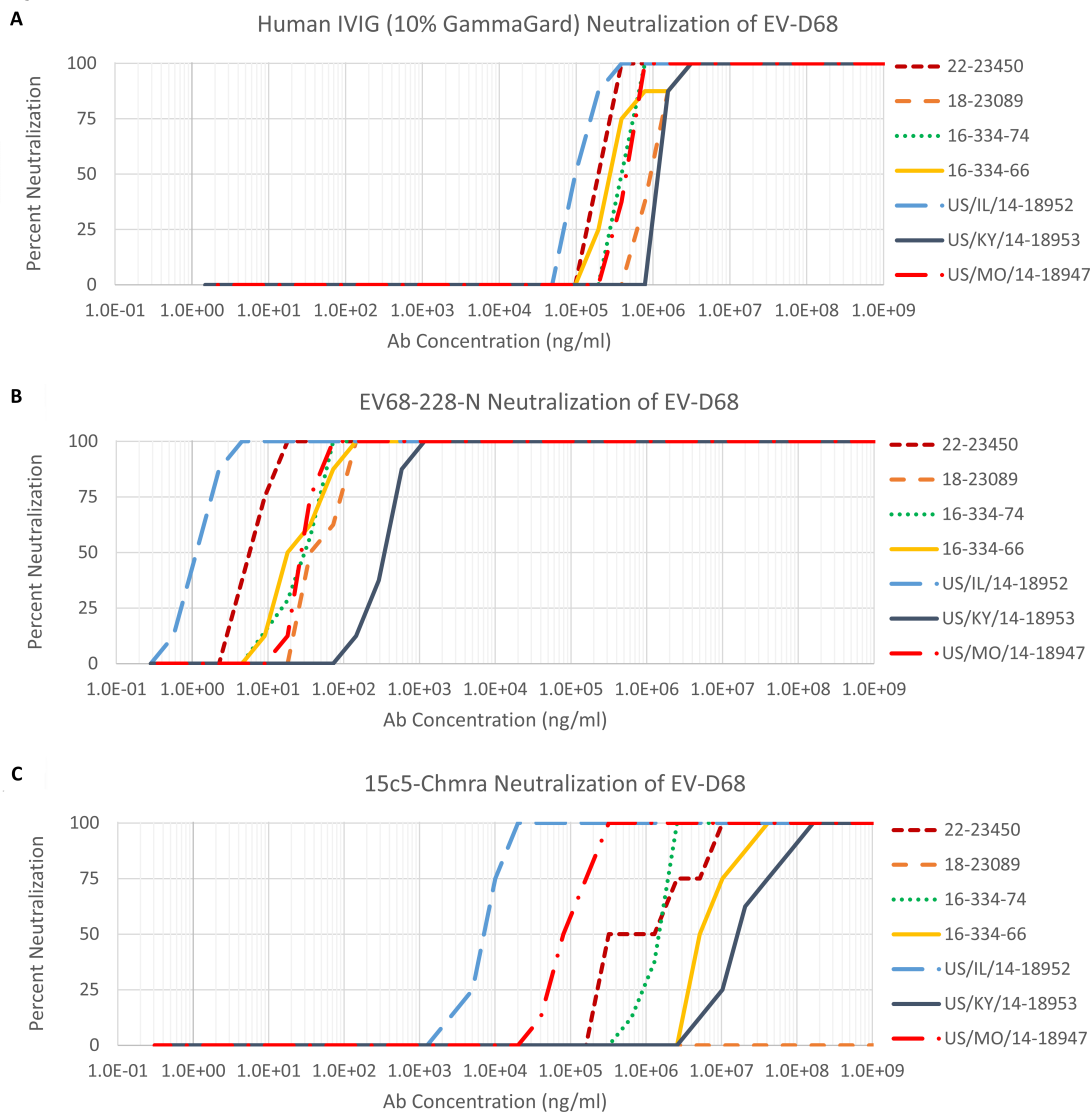
Neutralization assays showing the efficacy of pooled human IVIG (GAMMAGARD LIQUID), EV68-228-N mAb, and 15c5-Chmra mAb against 100 TCID_50_ EV-D68 from 2014 clade B1 isolate (US/MO/14-18947), 2014 clade D isolate (US/KY/14-18953), 2014 clade B2 isolate (US/IL/14-18952), 2 × 2016 epidemic isolates (2016-334-66 and 2016-334-74), a 2018 epidemic isolate (18-23089), and a 2022 epidemic isolate (22-23450). Antibody concentration on *x*-axis was calculated based on known starting concentrations of EV68-228-N and 15c5-Chmra IgG2B/k (19.05 mg/mL and 20.61 mg/mL, respectively), and for pooled human IVIG, the *x*-axis represents total polyclonal IgG (not just anti-EV-D68 antibody) in GAMMAGARD.

We further examined the neutralization potency of EV68-228-N against three 2014 EV-D68 isolates from clades D, B1, and B2, as well as viral isolates from the 2016, 2018, and 2022 epidemics. We also compared the *in vitro* efficacy of EV68-228-N mAb with 15c5-Chmra (a monoclonal antibody which we previously tested in the same model [8]) and against pooled human IVIG (there are anti-EV-D68 antibodies in the blood of nearly all adult humans). We found that the 2014 clade D isolate (US/KY/14-18953) and the 2022 viral isolate 22-23450 were the two isolates which are least susceptible to neutralization and conversely that the 2014 clade B2 isolate (US/IL/14-18952) was most susceptible to neutralization by all three antibodies (EV68-228-N mAb, 15c5-Chmra, and pooled hIVIG) tested here (Fig. 4). Notably, the IC_50_ for EV68-228-N against the least susceptible viral isolate (US/KY/14-18953 IC_50_ = 330 ng/mL) was 21× lower than the IC_50_ for 15c5-Chmra against the most susceptible viral isolate (US/IL/14-18952 IC_50_ = 6,961 ng/mL) (Table S1). The Hill coefficients for the data as well as the respective IC_90_, IC_95_, and IC_99_ values are shown in Table S1. We suggest that the increased efficacy of EV68-228-N at halting paralysis, compared to hIVIG and 15c5-Chmra (Fig. 1A), is due to the high neutralization potency of EV68-228-N (between 1 and 330 ng/mL) compared to hIVIG and 15c5-Chmra (Table S1). Additionally, we found that pooled human IVIG (GAMMAGARD LIQUID) neutralized all EV-D68 isolates within a narrow 14× dilution window (Fig. 4A), EV68-228-N neutralized all EV-D68 isolates within a larger 330× dilution window (Fig. 4B), and 15c5-Chmra failed to neutralize all isolates and had a dilution window greater than 100,000× (Fig. 4C). This data suggests that hIVIG and EV68-228-N are far more likely to retain neutralization potential against emerging EV-D68 viral isolates, compared to 15c5-Chmra.

## DISCUSSION

In our mouse model of EV-D68-induced AFM, paralysis/weakness is first observed in the left hindlimb (intramuscularly infected with EV-D68) approximately 4 days after infection. Loss of function in the left hindlimb occurs concurrent with loss of motor neurons from the left-side anterior horn of the lumbar enlargement. Weakness progressively worsens in a single limb, often resulting in complete paralysis of that limb, before spreading to other limbs and worsening in those limbs, reaching its nadir at approximately 6 days after initial onset of neurologic symptoms. The severity of neurologic symptoms in this mouse model varies from weakness in a single limb to complete quadriplegia. A similar progression of AFM symptoms is observed in human patients who present at the clinic with AFM. Following admission to the hospital, the neurologic symptoms of AFM patients often worsen over the course of hours to days and, in the most severe cases, can progress to complete quadriplegia (3). Here, we show that treatment with EV68-228-N monoclonal antibody can quickly halt the progression of paralysis (0.76 ± 0.21 days), preventing the spread of paralysis to the contralateral hindlimb and both forelimbs when administered to mice with nearly complete paralysis in a single limb. Treatment could not reverse paralysis which was present prior to antibody treatment, but this is not surprising since a loss of motor neurons from the ventral horn of the lumbar enlargement coincides with loss of function of the ipsilateral hindlimb, and antibody treatment would not be expected to restore lost motor neurons. Instead, it seems that monoclonal antibody treatment is preventing the loss of additional motor neurons by inhibiting viral spread to healthy motor neurons. This work suggests that the more quickly viral replication can be stopped, the greater the limb function that is retained.

We did not confirm the presence of EV68-228-N antibody in spinal cord tissue; however, given the significant reduction in viral titer and viral RNA in spinal cords from treated mice (reduced to the limit of detection) at both DPI-3 and DPI-6, it seems likely that monoclonal antibody is crossing the blood–brain barrier (BBB). This is consistent with work showing that small amounts of antibody can cross the BBB of healthy mice (14), and, given the high degree of neuronal death in our mouse model of AFM, it is likely that disruption to the BBB is allowing antibody to cross into the spinal cord. Moreover, the predictable spread of paralysis from the infected hindlimb to the contralateral hindlimb and then to the forelimbs suggests that EV-D68 is spreading from the infected muscle into the spinal cord and then spreading within the spinal cord to the ipsilateral hindlimb and eventually spreading up the spinal cord to the motor neurons which control the forelimbs. So, while we cannot eliminate the possibility that antibody treatment is neutralizing virus in the periphery alone and thereby reducing influx of virus into the CNS, our data strongly suggest that EV68-228-N antibody is crossing into the CNS and neutralizing virus within the spinal cord. Furthermore, we rarely detect infectious virus in the muscle of the contralateral hindlimb (21% at DPI-4, 11% at DPI-7, and 0% at DPI-10), and even when viral titer is detected, the TCID_50_ in the uninjected muscle is at least 100-fold lower than the TCID_50_ of the EV-D68-injected muscle. It is, therefore, unlikely that virus is spreading peripherally to the contralateral muscle and entering the CNS via retrograde transport from the contralateral hindlimb (as it does in the infected muscle [15]).

We confirm the findings of Vogt et al. (9) that EV68-228 is highly neutralizing against 2014 clades B1, B2, and D, and we further show that EV68-228-N can neutralize more recent 2016, 2018, and 2022 epidemic EV-D68 isolates at similarly low concentrations (1–330 ng/mL), without any evidence of emerging resistance or reduced sensitivity among newer viral isolates. We also compared the neutralization potency of EV68-228-N to 15c5-Chmra (another anti-EV-D68 neutralizing monoclonal antibody) and to pooled human IVIG and found that the weakest neutralization of EV68-228-N outperforms even the strongest neutralization of 15c5-Chmra or hIVIG. The difference in neutralization potency between these two monoclonal antibodies may reflect the difference in epitopes between the two. 15c5 binds at the threefold axis of symmetry, mimicking engagement by ICAM-5 (16), whereas 228 binds between depressions that form the canyon regions surrounding the fivefold axis and covers the classical picornavirus neutralizing immunogenic sites (NIms) NIm-IB (VP1 DE loop) and NIm-II (VP2 EF loop) (9). The 330× dilution window for EV68-228-N compared to the >100,000× dilution window for 15c5-Chmra, when comparing neutralization of seven viral isolates from different clades and across 9 years, suggests that the structure of threefold axis of symmetry (where 15c5 binds) may be more variable than the canyon surrounding the fivefold axis of symmetry (where EV68-228-N binds). The markedly greater dilution window for 15c5-Chmra compared to EV68-228-N suggests that EV68-228-N will retain efficacy against a much wider range of viral clades and years than 15c5-Chmra. Pooled human IVIG had a smaller dilution window than either of the monoclonal antibodies, suggesting that it will retain efficacy against a wider range of viral clades and years than either monoclonal antibody. This is not surprising since hIVIG is polyclonal and simultaneously binds many EV-D68 epitopes. When tested *in vivo*, however, hIVIG failed to halt the progression of paralysis as quickly as EV68-228-N, and the final paralysis scores of EV68-228-N-treated animals were significantly better than the final paralysis scores for hIVIG-treated animals. Since the maximum injectable volume of hIVIG (GAMMAGARD LIQUID) was used in the mouse experiments and since the neutralization potency of EV68-228-N was nearly 10,000× better than hIVIG, we conclude that EV68-228-N antibody would be a better therapeutic choice for treatment of AFM caused by 2014–2022 EV-D68 epidemic isolates. The data presented here, together with our previously published work on 15c5-Chmra and 11g1-Chmra, indicate that there is a correlation between the neutralization potency of the mAb and the final paralysis score of the treated animal. Treatment with mAbs with a greater neutralization potency (<1,000 ng/mL) will halt the progression of paralysis more quickly than treatment with mAbs with a lower neutralization potency (1,000–100,000 ng/mL) leading to a less severe final paralysis score.

Approximately 20% of mice showed signs of paralysis beginning 1–3 days (mean 1.30 ± 0.70 days) prior to meeting the minimum threshold for the initiation of treatment (minimum paralysis score of 1.5). A comparison of these mice showed that treatment with EV68-228-N significantly (*P* = 0.0003 repeated measures ANOVA) improved paralysis outcome (EV68-228-N-treated paralysis score = 2.5 ± .43; control treated paralysis score = 5.25 ± 1.17) even in this group. This data suggest that EV68-228-N can halt the progression of neurologic symptoms, even when antibody treatment is delayed by a day or more after the earliest signs of paralysis. However, our data strongly indicate that the more quickly treatment is administered, the better the patient outcome will be.

## MATERIALS AND METHODS

### Antibodies

Human IgG1 monoclonal antibodies, EV68-228-N and anti-HSV8 (control), were produced and provided by KBio Inc., as previously described (17). Both antibodies were expressed in and purified from a transgenic *N. benthamiana* variety deficient in xylosyl- and fucosyltransferase. The transgenic plant strain produces afucosylated glycoproteins without the non-mammalian xylose structures, making for a more human-like glycan structure (18). Agrobacterium cultures transformed with expression vector plasmids containing viral replicons specific for heavy and light chain components of the antibody are vacuum infiltrated into whole plants. Viral replicons drive the expression of the antibody subunits that are subsequently translated and assembled by the plant cellular machinery. Antibody is allowed to accumulate in the developing plant for 7 days, and the plant biomass is extracted through mechanical disintegration. The resulting homogenate is purified through a multistep chromatographic and filtration process yielding purified EV68-228-N or anti-HSV-8. Mouse–human chimeric 15C5-Chmra IgG2B/k monoclonal antibodies were constructed and provided by ZabBio. Sequences for the 15c5 variable heavy and variable light domains were retrieved from the Protein Data Bank deposit 6AJ7 (https://www.rcsb.org/structure/6AJ7), as published by Zheng et al. (16). These sequences were compared to conventional murine antibody domain structures using internal (Mapp) and external databases. In some cases, sequence modifications were made based on known conventions. These variable regions were synthesized to incorporate codon optimization for Mapp’s Nicotiana expression system and 5′/3′ cloning and restriction sites to accommodate expression vector assembly. Antibody purity was ≥95% and was assessed using size exclusion chromatography and the LabChip GXII protein characterization system. GAMMAGARD LIQUID for IV infusion (manufactured by Takeda Pharmaceuticals) was used for the positive control (hIVIG) and was a generous gift from Dr. Kevin Messacar at Colorado Children’s Hospital.

### Clinical treatment model

Litter size was kept constant at 10 animals per dam by mixing litters of the same age. Both male and female pups were used in these experiments, and there were no statistically significant differences in paralysis between the sexes. Swiss–Webster mouse pups were intramuscularly injected with 10 µL of EV-D68 at 10,000 TCID_50_/pup in the left quadriceps muscle when the average litter weight for pups was between 1.5g and 2.0g (postnatal days 1 and 2). Individual pups were uniquely marked and checked once daily for paralysis. When an individual animal was observed with a paralysis score that was greater than or equal to 1.5 (paralysis ≥ 2), the animal was intraperitoneally injected with 25 µL of antibody. The scientist who recorded weight and paralysis was blinded to the treatment groups. The paralysis score and weight for each animal were recorded daily. Uniquely marking each animal allowed data from the same animal to be tracked throughout the experiment, which allowed the experimenter to retrospectively assign each mouse to its appropriate treatment group (EV68-228-N, hIVIG, HSV8, or mock infected). Animals were tracked for a total of DPI-21.

### Paralysis scoring scale

Paralysis scores were assessed as previously published by Hixon et al. (10), with the exception that in rare instances, a score of 1.5 or 2.5 was used to better capture limb paralysis that was borderline between bins. Briefly, each limb was scored from 0 to 3, where 0 represents normal limb movement and 3 represents complete loss of function in the limb. The score from each limb was added together to give a total paralysis score up to 12 for a completely paralyzed, quadriplegic animal. An animal was sacked if its paralysis score was ≥11or if its weight dropped below 60% of the litter average.

### Viral stocks

The EV-D68 strain US/IL/14-18952, US/MO/14-18947, and US/KY/14-18953 (isolated in 2014) were purchased from BEI resources. The 2016-334-64, 2016-334-74, 2018-23089, and 2022-23450 viral isolates were a generous gift from the Wadsworth Center at the Department of Health in New York State. Virus was propagated once in RD cells, and cell debris was removed by centrifugation. All virus stocks were injected intramuscularly at 10,000× TCID_50_.

### TCID_50_ protocol

The spinal cord and quadriceps muscle tissue were dissected from mice, and the whole tissue was homogenized in 300 µL of ice-cold PBS. Twenty microliters of tissue lysate and serial 10-fold dilutions were added to the appropriate wells of a 96-well flat-bottomed cell culture plate containing rhabdomyosarcoma (RD) cells. Plates were incubated at 33°C for 7 days, and afterward, all wells were examined for signs of cytopathic effect. TCID_50_ values were calculated using the Reed–Muench method. All TCID_50_ values in this paper represent TCID_50_/mL even if it is not explicitly stated.

### Immunohistochemistry

Spinal cords were dissected from the spinal column in ice-cold PBS. They were fixed in 4% PFA for 24 hours and cryoprotected in 30% sucrose for 24–48 hours. The lumbar enlargement was dissected from the spinal cord and then frozen in optimal cutting temperature compound (OCT). Blocks were transversely sectioned at 20 micron thickness using Leica CM3050S cryotome prior to IHC staining. Antigen retrieval was performed, and the following primary antibodies were used: (i) anti-NeuN Rat mAb ab279297 from abcam used at 1:100 and (ii) anti-ChAT rabbit mAb (EPR 16590) ab178850 from abcam used at 1:50. A Leica confocal microscope with 10× objective was used to image spinal cord sections.

### Neutralization assays

Human rhabdomyosarcoma cells were grown in a 96-well flat-bottomed cell culture plate; 100× TCID_50_ EV-D68 virus (US/IL/14-18952, US/MO/14-18947, US/KY/14-18953, 2016-334-64, 2016-334-74, 2018-23089, or 2022-23450) was incubated for 1 hour with serial twofold dilutions of antibody (GAMMAGARD, EV68-228-N, or 15c5-Chmra) at 33C. After an hour of incubation, the media in the 96-well cell culture plate was replaced with media containing 100× TCID_50_ EV-D68 + antibody mixture, and plates were incubated at 33C for 7 days. All samples were run as 8× replicates. Afterward, all wells were examined for signs of cytopathic effect.

### Statistical analysis

JMP, Version 15.0.0 SAS Institute Inc., Cary, NC, 1989–2007, was used for all statistical calculations. We did not observe sex differences in survival or paralysis for any experiment. We did observe litter effects, so all treatments were administered to an equal number of pups within every litter, and a minimum of four litters were used to generate all data. For paralysis scores, any animal that died before completion of the 21-day experiment had its last recorded paralysis score carried forward throughout the remainder of the experiment. One animal died of unknown causes prior to the onset of paralysis and was removed from the experiment. For weights, if an animal died before the end of the experiment, then the previous 7 days of weight data were used to generate a trendline and estimate the expected weight on the missing days for this animal.

### IC_50_ calculations

IC_50_ values for Table S1 were calculated using online software (AAT Bioquest, Inc. [11 November 2024], Quest Graph IC50 Calculator, https://www.aatbio.com/tools/ic50-calculator).

### Real-time PCR protocol

Viral RNA was isolated from each fraction using a QIAmp Viral RNA Mini Kit (cat no. 52904) (see Wylie et al. [19]). The real-time PCR setup used reagents and master mix from the Bio-Rad iTaq Universal SYBR Green One-Step Kit (cat no. 1725150). Primer sequences were Fwd1-CAC(T/C)GAACCAGA(A/G)GAAGCCA, Rev1-CCAAAGCTGCTCTACTGAGAAA, and Rev2-CTAAAGCTGCCCTACTAAG(G/A)AA, (see Wylie et. al. [19]). Thermal cycling conditions were 50°C for 10 min, 95°C for 2 min, then 40 cycles of 95°C for 15 sec, and 60°C for 45 sec, followed by a melt curve analysis from 65°C to 95°C in 0.5°C increments. All samples had 3× technical replicates.

## SUPPLEMENTAL MATERIAL

Figure S1 mbio.03906-24-s0001.tiff Clinical model treatment paradigm.

Figure S2 mbio.03906-24-s0002.tiff RNA copy number in spinal cord and muscle.

Supplemental Legends mbio.03906-24-s0003.docx Legends for supplemental figures and table.

Table S1 mbio.03906-24-s0004.pdf Hill coefficients.

ASM does not own the copyrights to Supplemental Material that may be linked to, or accessed through, an article. The authors have granted ASM a non-exclusive, world-wide license to publish the Supplemental Material files. Please contact the corresponding author directly for reuse.
