# Supplementary figures and images for: EV68-228-N monoclonal antibody treatment halts progression of paralysis in a mouse model of EV-D68 induced acute flaccid myelitis
Source: mBio. 2025 Mar 24;16(4):e03906-24. doi: 10.1128/mbio.03906-24 (PMC11980581; doi:10.1128/mbio.03906-24)

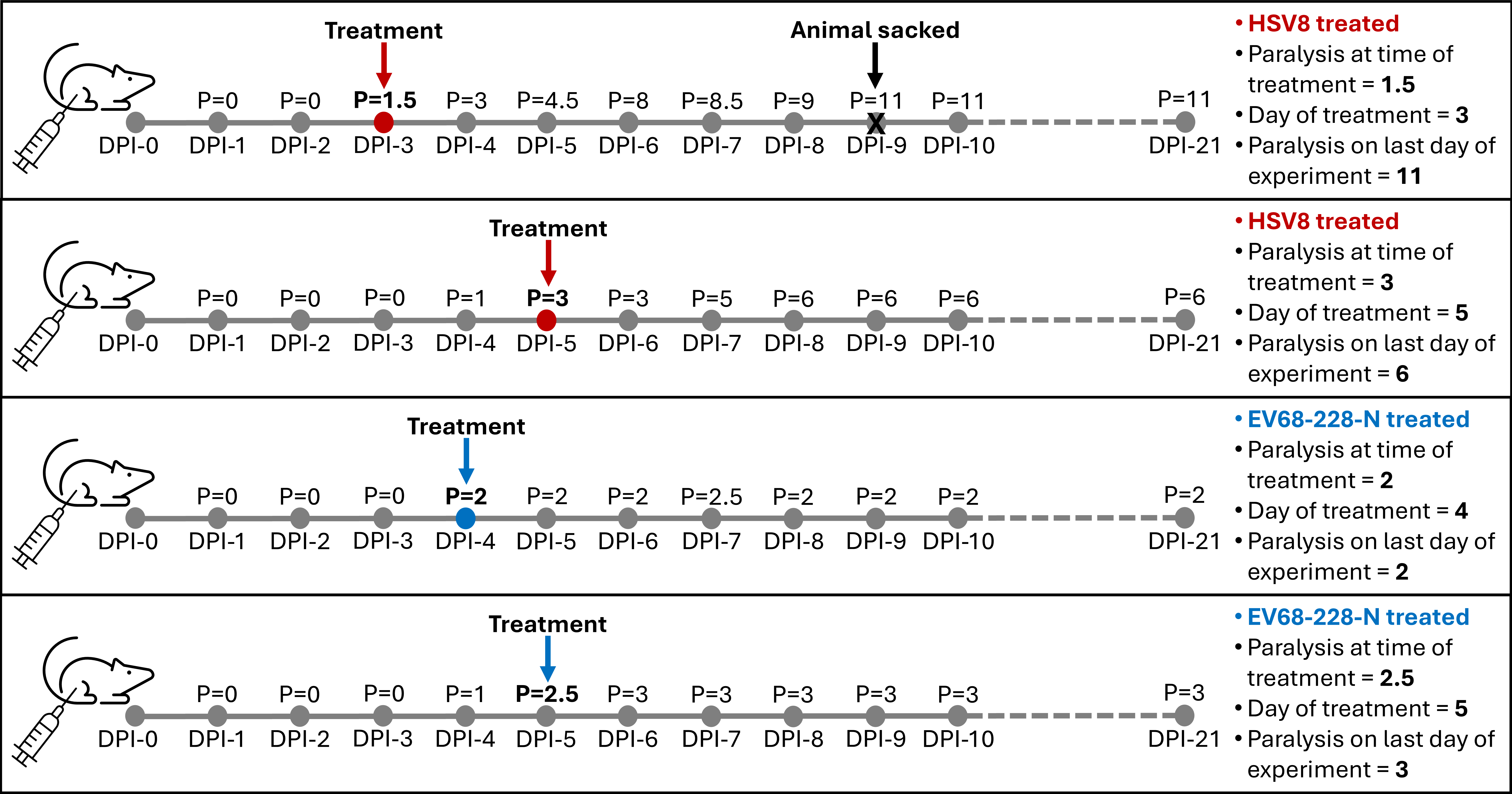

Supplement: Figure S1 — Clinical model treatment paradigm. [file mbio.03906-24-s0001.tiff]
