# Supplementary material for: EV68-228-N monoclonal antibody treatment halts progression of paralysis in a mouse model of EV-D68 induced acute flaccid myelitis
Source: mBio. 2025 Mar 24;16(4):e03906-24. doi: 10.1128/mbio.03906-24 (PMC11980581; doi:10.1128/mbio.03906-24)
Supplement: Supplemental Legends — Legends for supplemental figures and table. [file mbio.03906-24-s0003.docx]

Supplemental Figure 1 - Clinical model treatment paradigm. “DPI” represents days post infection and “p=0” represents paralysis score (with 0 being complete function in all limbs and 12 being complete quadriplegia). The threshold for treatment was set at 1.5 (the lowest score where paralysis could be identified with 100% confidence). An animal was sacked if its paralysis score reached or exceeded 11 (top row), and the final paralysis score was carried forward through the remainder of the experiment. The top two rows (red) show paralysis scores from individual animals treated with HSV8 monoclonal antibody on the day identified by the label “treatment” and an arrow. The bottom two rows show individual mice treated with EV68-228-N monoclonal antibody (blue dots) on DPI-4 or DPI-5 respectively.

Supplemental Figure 2 – Spinal cord (top) and muscle (bottom) samples collected from mice intramuscularly infected with EV-D68 US/IL/14-18952. Mock infected animals were

intramuscularly injected with sterile saline. Mice were intraperitoneally injected with 1mg/kg EV68-228-N or hIVIG or HSV8 (isotype control) or PBS (Placebo) when paralysis scores reached or exceeded 1.5 and tissue was collected 3 days post treatment. Quad muscle samples - There is a significant difference between isotype control and EV68-228-N (p=0.0004 one way ANOVA Dunnet’s method), hIVIG (p=0.0056 one way ANOVA Dunnet’s method), and mock (p=0.0011 one way ANOVA Dunnet’s method). Spinal Cord samples – There is a significant difference between isotype control and EV68-228-N (p=0.0167 one way ANOVA Dunnet’s method) and between mock and isotype control (p=0.0167 one way ANOVA Dunnet’s method). *p<0.05, **p<0.01, ***p<0.001. All data was log transformed prior to statistical analysis. Error bars represent SEM.

Supplemental Table 1 – Hill coefficients, IC90 , IC95, and IC99 values in ng/ml
