# Supplementary material for: EV68-228-N monoclonal antibody treatment halts progression of paralysis in a mouse model of EV-D68 induced acute flaccid myelitis
Source: mBio. 2025 Mar 24;16(4):e03906-24. doi: 10.1128/mbio.03906-24 (PMC11980581; doi:10.1128/mbio.03906-24)
Supplement: Table S1 — Hill coefficients. [file mbio.03906-24-s0004.pdf]

EV-D68 viral isolate	Human IVIG: Hill coefficient	EV68-228-N: Hill coefficient	15c5-Chmra: Hill coefficient
22-23450	-15.9994	-2.8892	-0.9866
18-23089	-3.8450	-2.2474	N/A
16-334-74	-15.9994	-2.2573	-5.0031
16-334-66	-2.8380	-1.7587	-2.4123
US/IL/14-18952	-3.8081	-2.8904	-2.9726
US/KY/14-18953	-13.8425	-3.1196	-1.622
US/MO/14-18947	-15.0474	-4.4490	-2.3157

EV-D68 viral isolate	Human IVIG IC_{90}	EV68-228-N IC_{90}	15c5-Chmra IC_{90}
22-23450	224,064	13	7,862,626
18-23089	1,602,205	122	> 1,000,000,000
16-334-74	448,128	74	2,181,558
16-334-66	616,221	80	14,914,590
US/IL/14-18952	179,014	2	14,578
US/KY/14-18953	1,591,124	667	70,055,066
US/MO/14-18947	467,647	46	223,691

EV-D68 viral isolate	Human IVIG IC_{95}	EV68-228-N IC_{95}	15c5-Chmra IC_{95}
22-23450	234,776	17	16,768,192
18-23089	1,945,881	171	> 1,000,000,000
16-334-74	469,554	103	2,532,963
16-334-66	801,828	123	20,329,881
US/IL/14-18952	217,822	3	18,744
US/KY/14-18953	1,679,373	848	111,047,308
US/MO/14-18947	491,456	54	308,875

EV-D68 viral isolate	Human IVIG IC_{99}	EV68-228-N IC_{99}	15c5-Chmra IC_{99}
22-23450	260,292	29	89,352,050
18-23089	2,989,245	355	> 1,000,000,000
16-334-74	520,585	214	3,523,030
16-334-66	1,434,440	314	40,300,689
US/IL/14-18952	336,012	5	32,660
US/KY/14-18953	1,892,063	1,440	307,242,925
US/MO/14-18947	548,436	79	630,025
